# A Comparison of Autorefraction and Subjective Refraction in an Academic Optometry Clinic

**DOI:** 10.7759/cureus.37448

**Published:** 2023-04-11

**Authors:** Parinee Kemchoknatee, Pornlada Sunlakaviset, Nattawat Khieokhoen, Thansit Srisombut, Duanghathai Tangon

**Affiliations:** 1 Ophthalmology, Rangsit University, Rajavithi Hospital, Bangkok, THA

**Keywords:** spherical equivalent, cylindrical power, spherical power, refraction, subjective refraction, autorefraction, autorefractor

## Abstract

Background: Refractive error is the most common cause of decreased visual acuity. Refractive measurement in adults consists of cycloplegic (objective) and manifest (subjective) refraction. Although the effectiveness of autorefraction is a crucial factor, there needs to be more information on its accuracy and precision on each autorefractor compared with subjective measurement in Thai patients.

Objective: To compare the accuracy and precision of the two autorefractors' findings in Rajavithi Hospital, OptoChek Plus, and TOMEY Auto Refractometer RC-5000, with each other and with those of the subjective method.

Materials & Methods: An observational study was conducted at the Ophthalmology clinic in Rajavithi Hospital from March 1, 2021, to March 31, 2022. All subjects were tested using the two autorefractors (OptoChek Plus and TOMEY Auto Refractometer RC-5000) and subjective refraction. One eye per subject was included in the study.

Results: Forty-eight patients (48 eyes) were enrolled in the study. The difference between spherical powers obtained by OptoChek and subjective refraction was not significantly different; however, there was a significant difference between those calculated by Tomey and the subjective method (p=0.77, p=0.04 respectively). The variations between cylindrical powers arrived at by the two autorefraction techniques and those calculated by the subjective method were significantly different (OptoChek and Tomey p-=0.01, p-value<0.001, respectively). In addition, 95% of the limit of agreement (95% of LOA) was low in the cylindrical measurement of each autorefractor compared with subjective refraction. (84.61%, 86.36%, respectively). No statistically significant difference between the spherical equivalent calculated by the two autorefractors and that of subjective refraction was observed in the present study (OptoChek: p-value=0.26 and Tomey: p-value=0.77).

Conclusions: There was a clinically significant difference between the cylindrical power calculated by the two autorefractors and those obtained from subjective refraction. Patients with high astigmatism should be monitored closely when measured by autorefractors, as there can be a slightly lower agreement between objective and subjective refraction.

## Introduction

In a non-accommodated state, emmetropia is defined as the state of vision resulting from the coordination between the cornea and lens in focusing the rays onto the retina. In ametropia, certain rays may reach the retina's focal point either anteriorly or posteriorly, resulting in a change in the focal point location on the retina. Myopia is an eye disorder where light rays focus anterior to the retina in a non-accommodated state. When light rays focus posterior to the retina in that state, it is defined as hyperopia. Astigmatism is an ophthalmologic condition characterized by two or more focal lengths with no focus at the retina's focal point due to different refractive powers in each axis [1].

In 2010, a WHO survey reported that uncorrected refractive error is the most prevalent cause of low vision and the second most common cause of blindness [2]. Corrections of refractive error can be treated with various methods, including corrective glasses, contact lenses, and refractive surgery; corrective glasses are the most effective [3].

Objective refractive measurement in adulthood consists of retinoscopy and autorefraction. An autorefractor is a well-established measurement tool controlled by a computer-based program. It is used to calculate the estimated refractive power and combine it with the results of a subjective test - a conservative refractive measurement with a trial lens, or phoropter, placed anterior to the subject's eyes, followed by a refraction chart reading, all intending to determine the best corrected visual acuity [4].

In 2004, Konrad Pseudovs et al. studied the accuracy and precision of the refractive values calculated by two autorefractors, Nidek ARK-700A, and Topcon KR-8000, compared with those derived from previous corrective glasses after subjective measurement. The result was not clinically significant between the spherical equivalent values obtained using the subjective method and those obtained by autorefraction. No comparison was made between the measurements derived by the two autorefractors in their study [5].

The study aimed to compare the accuracy and precision of refractive values obtained by the two autorefractors used in Rajavithi Hospital, OptoChek Plus, and TOMEY Auto Refractometer RC-5000, those calculated using the subjective method.

## Materials and methods

The study was observational in design, with examination occurring between March, 1,2021, to March 31, 2022. All participants were refracted by two autorefractors: OptoChek Plus and TOMEY Auto Refractometer RC-5000 (OptoChek® Plus Auto Refractor+Keratometer, Reichert, USA; RC-5000 Auto Ref-Keratometer, TOMEY, DE). Subjects were prospectively recruited. Inclusion criteria were at least 18 years old and an uncorrected distance visual acuity (UCDVA) in both eyes of better than 20/200. The exclusion criteria were as follows: any ocular pathology, including any condition known to interfere with autorefractor performance, e.g., asteroid hyalosis; any other abnormality, including amblyopia and strabismus; any previous ocular surgery; or insufficient mental ability to comply with subjective refraction requirements with confidence. Only one eye per subject was included in the study (normally the right unless only the left satisfied the inclusion and exclusion criteria).

Manifest refraction was measured using subjective refraction only. The ophthalmologist (N.K.) conducted autorefraction while another optometrist (A.S.) performed subjective refraction, masked to the refractive error results derived from which autorefractor. However, she was unmasked to that of previous spectacles. Subjective refraction was performed using a trial frame in which loose lenses could be inserted so that the lens with the best refractive endpoint, instead of the eye, was separated by a vertex distance of 12 mm.

Careful subjective refraction was undertaken by determination of the best vision sphere and cylinder using Jackson's cross-cylinder technique. Changes in cylinder power were compensated for by adjustment of sphere power, but all such compensations were double-checked subjectively. Each eye was refracted monocularly, followed by binocular balancing. The final spherical power was defined as the highest plus value or the lowest minus value that gave the best visual acuity. All refractive measurements were carried out without cycloplegia, and manifest refraction was recorded to the nearest 0.25 diopters sphere (DS), 0.25 diopters cylinder (DC), and 5°.

The OptoChek Plus works according to Scotopic Pupil Size (SPS) and Photopic Pupil Size (PPS) modes by adjusting illumination to accommodate different lighting conditions. Accuracy and continuity are achieved by wide-ranging measurements of spheres (-30 to+22 Diopters [D]), cylinders (0 to±10 D), and axes (0º to 180º), with an accuracy set to 0.12/0.25 D for power and 1° for axis. This device is capable of measuring pupils as small as 2.0 mm.

TOMEY Auto Refractometer RC-5000 operates with electronically controlled movement. Handling the RC-5000 requires alignment of the optical head towards the patient's eye. It accurately and continuously measures spheres (-25 to+22 D; at Vertex Distance=12 mm), cylinders (0 to±10 D; at Vertex Distance=12 mm), and axes (0° to 180°), with precision set to 0.01/0.12/0.25 D for power and 1° for axis. It can measure pupils as small as 2.2 mm.

Previous spectacle corrections were also recorded for the subjective refraction process. This was to determine whether autorefraction or previous spectacles made a better starting point for subjective refraction (i.e., which was closer to the subjective refraction endpoint). The results of the subjective method, along with the autorefraction and previous spectacle correction data, were stored in a spreadsheet and converted into spherical equivalents (sphere+cylinder/2) to calculate differences. These differences were positive if the autorefraction measurement was more hyperopic (less myopic) than the subjective refraction and negative if the autorefraction results were more myopic (less hyperopic) than those of subjective refraction.

Ethical approval

The study adhered to the tenets of the Declaration of Helsinki and was approved by Rajavithi Hospital Research Ethics Committee (Certificate number: 064/2564). All the patients provided written informed consent before data were collected.

Statistical analysis

Continuous data such as age and mean best-corrected distance visual acuity (BCDVA) were expressed as mean plus or minus SD. Categorical data such as gender and refractive error type were expressed as percentages. The differences between the autorefractive and subjective values of sphere, cylinder, and spherical equivalent (SE) were analyzed using Student's t-test. The difference between OptoChek and subjective refraction and that of Tomey and subjective refraction regarding sphere, cylinder, and SE were computed by paired t-test. Agreement between autorefraction and subjective measurement was expressed with 95% of the limit of agreement in a Bland-Atman plot. Statistical significance was set at a p-value less than 0.05, and all analyses were performed with SPSS version 25 (IBM Corporation, Armonk, NY, USA).

## Results

The total sample consisted of 48 subjects (48 right eyes) whose mean age was 53.02 ± 12.14 years, and 68.75% of whom were female (Table 1). Their mean BCDVA was 0.35±0.29 LogMAR (Logarithm of the Minimal Angle of Resolution). Subjective spherical power was +0.23±2.29 D, subjective cylinder power was -0.81±1.05 D; and subjective spherical equivalent refractive error was -0.18±2.39 D. OptoChek Plus found spherical power +0.26±2.46 D, cylinder power -1.01±1.09 D, and spherical equivalent refractive error -0.33±2.52 D. The TOMEY Auto Refractometer RC-5000 calculated spherical power +0.40±2.65 D, cylinder power, -1.09±1.08 D, and subjective spherical equivalent refractive error -0.15±2.76 D (Table 2).

**Table 1:** Baseline demographic data.* * Refractive errors are categorized based on measurements by the subjective method. BCDVA; Best-corrected distance visual acuity at 4-meter distances. CI; confidence interval.

Variable (%)	Total (48 eyes, 48 patients)
Age (years old) – mean (±SD)	53.02 (12.14)
Gender – Female	33 (68.75)
Type of Refractive errors	
Myopic astigmatism	13 (27.08)
Hyperopic astigmatism	30 (62.5)
Emmetropia	5 (10.42)
BCDVA – mean (±SD)	0.35 (0.29)

**Table 2:** Baseline refractive data. * * Spherical equivalent refraction (SER) = $\frac{\left [Sphere power+(Cylinder power)\right ]}{2}$\).

mean (±SD)	OptoChek	Tomey	Subjective
Sphere power	0.26 (2.46)	0.40 (2.65)	0.23 (2.29)
Cylinder power	-1.01 (1.09)	-1.09 (1.08)	-0.81 (1.05)
Spherical equivalent refraction^*^	-0.33 (2.52)	-0.15 (2.76)	-0.18 (2.39)

Spherical power analysis

**Figure 1:**
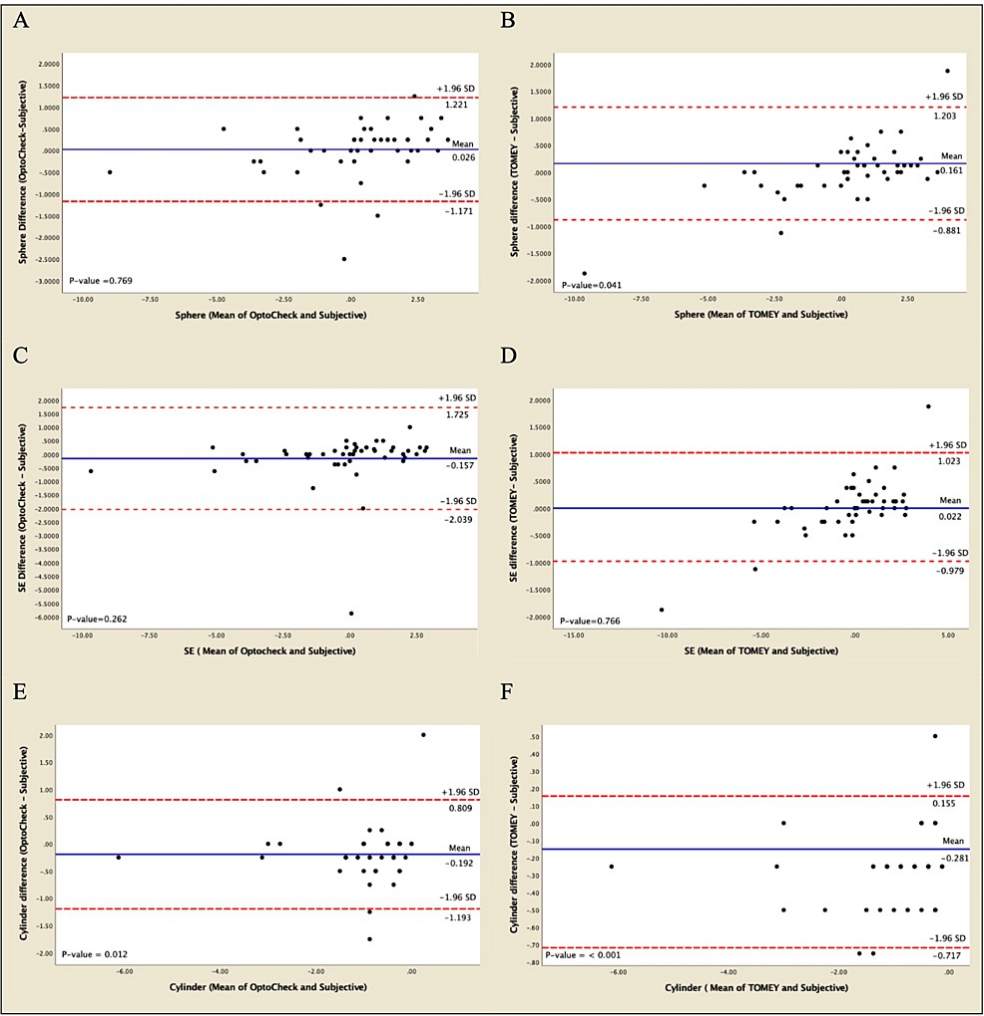
Bland–Altman Plots Evaluating the difference between measurements from each autorefractor and subjective refraction.* * The agreement between subjective and autorefraction Sphere (A, B) for A: the OptoChek®, the lines indicate mean agreement (solid blue line at 0.026 Diopter) and the 95% limits of agreement (dashed red lines at -1.171 and 1.221 Diopter), and B: the Tomey, the lines indicate mean agreement (solid blue line at 0.161 Diopter) and the 95% limits of agreement (dashed red lines at -0.881 and 1.203 Diopter). Spherical equivalent (C, D) for C: OptoChek®, the lines indicate mean agreement (solid blue line at -0.157 Diopter) and the 95% limits of agreement (dashed red lines at -2.039 and 1.725 Diopter), and D: Tomey, the lines indicate mean agreement (solid blue line at 0.022 Diopter) and the 95% limits of agreement (dashed red lines at -0.979 and 1.023 Diopter). Cylinder (E, F) for E: OptoChek®, the lines indicate mean agreement (solid blue line at -0.192 Diopter) and the 95% limits of agreement (dashed red lines at -1.193 and 0.809 Diopter), and F: Tomey, the lines indicate mean agreement (solid blue line at -0.281 Diopter) and the 95% limits of agreement (dashed red lines at -0.717 and 0.155 Diopter).

In terms of spherical power, there was a mean difference between the values measured by OptoChek Plus and subjective refraction of +0.25±2.36 D (Table 3), which gave 95% limits of agreement of -1.171 to +1.221 D (93.18%) (Fig 1A), while the mean difference between the TOMEY RC-5000 values and the subjective technique was +0.32±2.46 D with 95% limits of agreement of -0.881 to +1.203 D (93.18%) (Fig 1B). The mean difference between each platform [(OptoChek-subjective)-(Tomey-subjective)] was -0.07±0.36 D (p-value=0.201) (Table 4). The difference between spherical powers obtained by OptoChek and the subjective method was not significantly different; however, the values obtained by Tomey were significantly different from those of the subjective technique [OptoChek: p-value=0.77 (95% CI -0.15 to +0.20) and Tomey: p-value = 0.04 (95% CI +0.01 to +0.32)] (Table 5).

**Table 3:** The mean difference between measurements from each autorefractor and subjective refraction.* * p value; Paired samples T test. OptoChek-subj; difference between values obtained from OptoChek® Plus and subjective measurement. Tomey-subj; difference between values calculated by Tomey RC-5000 Auto Ref/K and subjective measurement. † Spherical equivalent refraction (SER) = $\frac{Sphere power+Cylinder power}{2}$.

mean (±SD)	Optochek-subj	Tomey-subj	p value
Sphere power	0.25 (2.36)	0.32 (2.46)	0.201
Cylinder power	-0.91 (1.04)	-0.95 (1.06)	0.261
Spherical equivalent refraction^†^	-0.25 (2.41)	-0.16 (2.57)	0.312

**Table 4:** Comparison of the mean difference between subjective measurement and each platform of autorefractor.* * p value; Paired samples T-test.

mean (±SD)	Diff OptoChek-subj – Tomey-subj	p value
Sphere power	-0.07 (0.36)	0.201
Cylinder power	0.04 (0.27)	0.261
Spherical equivalent refraction	-0.09 (0.61)	0.312

**Table 5:** One sample T-test for Equivalence.* * OptoChek-subj; difference between values derived from OptoChek® Plus and subjective measurement. Tomey-subj; difference between values obtained from Tomey RC-5000 Auto Ref/K and subjective measurement. CI; confidence interval. Spherical equivalent refraction (SER) = $Sphere power+\frac{Cylinder power}{2}$.

	p value	95% CI
Sphere power OptoChek-Subj	0.77	-0.15 – 0.20
Sphere power Tomey-Subj	0.04	0.01 – 0.32
Spherical equivalent OptoChek-Subj	0.26	-0.44 – 0.12
Spherical equivalent Tomey-Subj	0.77	-0.13 – 0.17
Cylinder power OptoChek-Subj	0.01	-0.34 – -0.04
Cylinder power Tomey-Subj	<0.001	-0.35 – -0.22

Cylindrical Power Analysis

The mean difference between cylindrical powers derived from the OptoChek Plus group and those found by subjective refraction was -0.91±1.04 D (Table 3), with 95% limits of agreement of-1.193 to +0.809 D (84.61%) (Fig 1E), while the mean difference between the TOMEY RC-5000 and subjective refraction was -0.95±1.06 D with 95% limits of agreement of -0.717 to +0.155 D (86.36%) (Fig 1F). The difference between the mean differences of each platform [(OptoChek-subjective)-(Tomey-subjective)] was +0.04±0.27 D (p-value=0.261) (Table 4). The differences between cylindrical powers obtained by the two autorefractors and the subjective method were significantly different [OptoChek: p-value=0.01 (95% CI -0.34 to -0.04) and Tomey: p-value < 0.001 (95% CI -0.35 to -0.22)] (Table 5).

Spherical Equivalent Analysis

The difference between the spherical equivalent values obtained by OptoChek Plus and the subjective evaluation (SE) was -0.25±2.41 D (Table 3), which gave 95% limits of agreement of -2.039 to +1.725 D (97.82%) (Fig 1C), while the TOMEY RC-5000 calculations had a mean difference of -0.16±2.57 D and 95% limits of agreement of -0.979 to +1.023 D (97.82%) (Fig 1D). The difference between the mean differences of each platform [(OptoChek-subjective)-(Tomey-subjective)] was -0.09±0.61 D (p-value=0.312) (Table 4). The difference between SE found by each autorefractor and the subjective method was not significantly different [OptoChek: p-value=0.26 (95% CI -0.44 to +0.12) and Tomey: p-value=0.77 (95% CI -0.13 to +0.17)] (Table 5).

## Discussion

Visual acuity (VA) is a crucial factor in ophthalmology, and its assessment is the first procedure performed after the history-taking step. Sometimes, a more specific test for VA, "best-corrected distance visual acuity" or "BCDVA," is used for diagnosis. The importance of VA in ophthalmologic differential diagnosis and treatment must be balanced. The outpatient department could save significant time if the VA and refractive value measurement durations were reduced.

Our study focused on improving the precision of VA measurement to achieve shortened measurement periods. We compared the refractive values obtained by two autorefractors (OptoChek Plus and TOMEY Auto Refractometer RC-5000) and those calculated using the subjective refraction method.

The results of previous studies comparing calculations of one autorefractor with those of another, as well with those made by the subjective method, have shown no statistically significant difference, except for the results of Justyna Wosik et al., in which the refractive values obtained from autorefraction and the subjective method were found to be different [6].

Our results showed no statistically significant difference in the autorefractors' spherical equivalent (SE) values and those of the subjective method. The spherical power obtained from TOMEY was significantly different from that derived from the subjective method, while the 95% CI ranged from +0.01 to +0.32 D, showing no clinical significance. In contrast, the differences between the cylindrical power values obtained by each autorefractor and those of the subjective method were statistically significantly different.

When comparing the mean difference between the autorefractors and the subjective method, it was observed that the cylindrical powers in minus received from the two autorefractors were higher than those obtained from the subjective method by about 1D (OptoChek 0.91 D and TOMEY 0.95 D). The spherical powers received from OptoChek and TOMEY were 0.25 D, and 0.32 D, respectively. The spherical equivalent (SE) values received from OptoChek and TOMEY were -0.25 D and -0.16 D, respectively, with a difference of about 0.25 D. Therefore, in this study, the spherical powers and the spherical equivalent (SE) values of the autorefractors were similar to those of the subjective method. To the best of our knowledge, the current series is the first clinical study comparing refraction results from each autorefractor with subjective measurement in Thai populations.

There were limitations in our study that should be acknowledged. First, its small number of patients (due to being conducted during the COVID-19 era) was a significant limitation. Second, astigmatism should be compared with Fourier or vector analysis of differences to consider the magnitude and direction of two cylinders when calculating their difference [7-13]. In the present study, we reported only the power representing subjective refraction and autorefraction astigmatism. Third, the two autorefractors used may not be available in other institutes.

## Conclusions

In conclusion, our work demonstrated that spherical power and SE values obtained from the two autorefractors and those calculated using the subjective method were not clinically significantly different; however, patients with high astigmatism should be observed when being measured by autorefractors, as there may be a slightly lower accordance between auto and subjective refraction. Further research in this clinic is warranted with a larger group of subjects, including subjects of a broader age range, especially children. There was a clinically significant difference between the cylindrical power calculated by the two autorefractors and those obtained from subjective refraction.
